# Association of computed tomography-derived body composition with surgical and oncologic outcomes in periampullary adenocarcinoma

**DOI:** 10.1007/s00464-026-12601-2

**Published:** 2026-02-06

**Authors:** Won-Gun Yun, Youngmin Han, Inhyuck Lee, Go-Won Choi, Younsoo Seo, Yoon Soo Chae, Young Jae Cho, Hye-Sol Jung, Wooil Kwon, Jin-Young Jang, Joon Seong Park

**Affiliations:** 1 Department of Surgery and Cancer Research Institute, Seoul National University College of Medicine, 101 Daehak-ro, Jongno-gu, Seoul, Republic of Korea; 2 Department of Surgery, Seoul Metropolitan Government-Seoul National University Boramae Medical Center, Seoul National University College of Medicine, 20, Boramae-ro 5-gil, Dongjak-gu, Seoul, Republic of Korea

**Keywords:** Periampullary cancer, Pancreaticoduodenectomy, Sarcopenic obesity, Sarcopenia, Minimally invasive surgery, Body mass index

## Abstract

**Background:**

Although the clinical efficacy of body composition assessment has been explored in many other cancer types, few studies have focused on periampullary cancer. Furthermore, despite the global rise in minimally invasive pancreaticoduodenectomy (PD), its safety and feasibility in patients with sarcopenic obesity remain unclear. We aimed to investigate the impact of body composition assessment on outcomes after PD and to evaluate the safety of minimally invasive PD in patients with sarcopenic obesity.

**Methods:**

Between 2015 and 2023, we included patients who underwent PD performed by surgeons who had surpassed the learning curve and were histologically diagnosed with periampullary cancer. Body composition was assessed using the axial images at the L3 vertebra level obtained from contrast-enhanced computed tomography.

**Results:**

Among 717 patients, 558 (77.8%) underwent open PD and 159 (22.2%) received minimally invasive PD. In multivariate logistic regression analysis, sarcopenic obesity (odds ratio [95% confidence interval]: 1.84 [1.23–2.77]; *P* = 0.003) was identified as an independent predictor of complications after PD, whereas high body mass index (≥ 25 kg/m^2^) and sarcopenia were not. Among patients with sarcopenic obesity, the open and minimally invasive PD groups demonstrated comparable short-term surgical outcomes—including complication rates—as well as oncologic outcomes such as the number of harvested lymph nodes and R0 resection rates.

**Conclusion:**

This study demonstrated that computed tomography-derived body composition variables could be helpful in predicting complications after PD. Additionally, minimally invasive PD could be carefully performed by experienced surgeons even in patients with sarcopenic obesity.

**Supplementary Information:**

The online version contains supplementary material available at 10.1007/s00464-026-12601-2.

Periampullary adenocarcinoma comprises four types of cancer: pancreatic head ductal adenocarcinoma, distal cholangiocarcinoma, ampullary adenocarcinoma, and duodenal adenocarcinoma [1]. Although each disease has distinct clinical and molecular characteristics, they are collectively referred to as periampullary adenocarcinoma due to their anatomic proximity in tissue of origin [2, 3]. While treatment approaches differ significantly among the types of cancer, the primary treatment for resectable disease is surgical resection followed by adjuvant treatment [4–7]. Surgery remains the only treatment modality with curative potential, and all four types of cancer are typically managed using pancreaticoduodenectomy (PD).

PD is considered one of the most complex procedures in general surgery, associated with substantial morbidity. Some high-volume, specialized centers have successfully reduced perioperative mortality rates to less than 3% through advances in perioperative management protocols [8]. However, several evolving factors may influence surgical outcomes. First, the global prevalence of obesity has risen to epidemic proportions in recent decades, partly due to increasingly westernized diets. By 2030, more than one-third of the global population is expected to be affected by overweight or obesity [9, 10]. Obesity is widely recognized as a risk factor for increased postoperative complications and poorer long-term outcomes [11]. Recent studies in other cancer types have suggested that assessing body composition—including skeletal muscle and visceral fat—may be more informative in predicting short- and long-term postoperative prognosis than obesity defined solely by body mass index (BMI). Nevertheless, studies in periampullary cancer remain limited [12, 13]. Second, minimally invasive PD has gained popularity in recent years, with an increasing number of institutions offering this approach. Despite its anatomic complexity and high risk of morbidity and mortality, minimally invasive PD has gradually been adopted [14, 15]. After the learning curve has been overcome, minimally invasive PD is considered as safe as open PD. However, limited evidence is available regarding its safety in patients with obesity or high visceral fat levels [16, 17]. Therefore, we aimed to evaluate the predictive value of obesity and body composition on surgical outcomes, and to assess the safety of minimally invasive PD in patients at high risk for surgical morbidity.

## Materials and methods

### Patient cohort

This single-center retrospective cohort study was approved by the Institutional Review Board of Seoul National University Hospital (H-2504-152-1634). This study was conducted in accordance with the 1975 Declaration of Helsinki and its subsequent revisions.

As our institution has performed minimally invasive PD since 2015, consecutive patients who underwent PD and were histologically diagnosed with periampullary adenocarcinoma (pancreatic head ductal adenocarcinoma, distal cholangiocarcinoma, ampullary adenocarcinoma, and duodenal adenocarcinoma) between 2015 and 2023 were included. Patients who received chemotherapy or radiotherapy prior to surgery (*n* = 279), and those with insufficient clinical information (*n* = 12), were excluded. Ultimately, 969 patients were enrolled, of whom 717 underwent surgery after the respective learning curves has been surpassed (Fig. S1). The learning curve for open PD was defined as 60 cases and for minimally invasive PD as 40 cases [18, 19].

### Surgical procedures

At our institution, pylorus-preserving PD was the standard procedure. However, classical PD with gastrectomy was performed in limited cases, such as those involving tumor infiltration or duodenal ischemia. Lymph nodes—specifically those surrounding the hepatoduodenal ligament and the pancreatic head—were removed without nerve dissection around the hepatic artery or superior mesenteric artery, in accordance with findings from a randomized controlled trial [20]. Extended lymph node dissection was permitted only when lymph node metastasis beyond the typical dissection range was strongly suspected on preoperative imaging. Following resection, a two-layer, end-to-side, duct-to-mucosa pancreaticojejunostomy was performed using the modified Blumgart method, with placement of an internal stent. For open surgery, a midline incision from the xiphoid process to the right of the umbilicus was used. In minimally invasive PD, five to six ports were inserted; the resection phase was conducted laparoscopically, while reconstruction was completed using a robotic platform (Da Vinci Xi), as previously described [21, 22].

### Postoperative evaluation

Contrast-enhanced abdomen–pelvis computed tomography was performed on postoperative day 4 to evaluate surgical morbidity. Complications classified as Clavien–Dindo grade III or higher were considered clinically significant [23]. Postoperative pancreatic fistula was graded according to the International Study Group of Pancreatic Surgery, with grades B and C regarded as clinically relevant [24]. Adjuvant chemotherapy was recommended in accordance with the National Comprehensive Cancer Network guidelines for each cancer type [4–7].

After discharge, patients were monitored using contrast-enhanced abdomen–pelvis computed tomography in combination with serum tumor marker testing. Recurrence was defined as radiologic evidence of recurrent disease.

### Data collection

Clinicopathological data, including demographic information, were collected. BMI was measured at the time of hospital admission for surgery, and obesity was defined as BMI ≥ 25 kg/m^2^ according to the World Health Organization criteria for the Asia–Pacific region. To assess body composition, axial images at the L3 vertebra level were extracted from contrast-enhanced abdomen–pelvis computed tomography performed within 1 month prior to surgery. These images were segmented into inner and outer compartments based on the muscle boundary using MATLAB (The MathWorks Inc., Natick, MA). Skeletal muscle area (cm^2^) and visceral fat area (VFA; cm^2^) were measured. Definitions of sarcopenia and sarcopenic obesity were based on previous studies. Sarcopenia was defined as a skeletal muscle index (SMI = skeletal muscle area/height^2^) < 55 cm^2^/m^2^ for males and < 39 cm^2^/m^2^ for females [25]. Sarcopenic obesity was defined as VFA/SMI > 2.5 m^2^ [26].

### Statistical analysis

Continuous variables were expressed as medians with interquartile ranges, and categorical variables were reported as counts with percentages. The Mann–Whitney *U* test was used to compare continuous variables, while the *χ*^2^ test or Fisher’s exact test was applied to compare categorical variables.

To identify independent predictors of postoperative complications, logistic regression models were employed. Results were presented as odds ratios (ORs) with 95% confidence intervals (CIs). Cox proportional hazards regression models were used to evaluate predictors of recurrence, with results reported as hazard ratios (HRs) and 95% CIs. In both logistic and Cox regression analyses, univariate models included all clinically relevant variables identified from the literature and clinical experience. Multivariate models included only those variables with *P* < 0.25 in the univariate analysis. Following logistic regression, clinical variables were compared according to surgical approach in subgroups stratified by body composition factors identified as independent predictors of postoperative complications.

*P* values less than 0.05 were considered statistically significant, while *P* values between 0.05 and 0.10 were considered marginally significant. All statistical analyses were performed using R software (version 4.4.0; R Foundation for Statistical Computing).

## Results

### Baseline characteristics

Clinical characteristics of the entire cohort are presented in Table 1. There were 593 (61.2%) men and 376 (38.8%) women, with a median age of 68 years. Among the 969 patients, 706 (72.9%) underwent open PD, and 263 (27.1%) received minimally invasive PD. High BMI, sarcopenia, and sarcopenic obesity were observed in 269 (27.8%), 496 (51.2%), and 386 (39.8%) patients, respectively. There were no significant differences in these variables between the open PD and minimally invasive PD groups. Patients with pancreatic and distal common bile duct cancer underwent open PD more frequently than minimally invasive PD (*P* < 0.001). The minimally invasive PD group had a significantly longer median operation time than the open PD group (315 versus 285 min, *P* < 0.001). Median intraoperative blood loss was comparable between groups (400 versus 400 mL, *P* = 0.273). Complication rates were marginally higher in the minimally invasive PD group than in the open PD group (25.1% versus 10.6%, *P* = 0.090), whereas the median length of hospital stay was significantly shorter in the minimally invasive PD group (8 versus 11 days, *P* < 0.001).

**Table 1:** Clinical characteristics according to the operation method in entire cohort

Parameter	Total	Open	Robot	*P*
N (%)	969 (100.0)	706 (72.9)	263 (27.1)	
Age (years)^a^	68 (61–74)	68 (61–75)	66 (61–74)	0.140
Sex				0.270
Male	593 (61.2)	440 (62.3)	153 (58.2)	
Female	376 (38.8)	266 (37.7)	110 (41.8)	
ASA				0.183
I/II	849 (87.6)	612 (86.7)	237 (90.1)	
III/IV	120 (12.4)	94 (13.3)	26 (9.9)	
BMI (kg/m^2^)				0.571
< 25	700 (72.2)	506 (71.7)	194 (73.8)	
≥ 25	269 (27.8)	200 (28.3)	69 (26.2)	
Sarcopenia	496 (51.2)	369 (52.3)	127 (48.3)	0.303
Sarcopenic obesity	386 (39.8)	285 (40.4)	101 (38.4)	0.630
Diagnosis				< 0.001
Pancreas	345 (35.6)	273 (38.7)	72 (27.4)	
dCBD	341 (35.2)	263 (37.2)	78 (29.7)	
AoV	246 (25.4)	142 (20.1)	104 (39.5)	
Duodenum	37 (3.8)	28 (4.0)	9 (3.4)	
Operation time (min)^a^	298 (250–352)	285 (235–340)	315 (280–373)	< 0.001
Blood loss (mL)^a^	400 (250–600)	400 (250–600)	400 (220–640)	0.273
Harvested LN	18 (12–24)	18 (13–24)	17 (12–24)	0.134
LN metastasis	495 (51.1)	394 (55.8)	101 (38.4)	< 0.001
Resection margin				0.003
R0	737 (76.1)	519 (73.5)	218 (82.9)	
R1	232 (23.9)	187 (26.5)	45 (17.1)	
Complication^b^	206 (21.3)	140 (19.8)	66 (25.1)	0.090
CR-POPF	109 (11.2)	75 (10.6)	34 (12.9)	0.371
Hospital stays (days)	10 (8–15)	11 (9–18)	8 (7–11)	< 0.001
Adjuvant chemotherapy^c^	423 (85.5)	331 (84.0)	92 (91.1)	0.101

Values in parentheses are percentages unless indicated otherwise

*ASA* American Society of Anesthesiologists, *BMI* body mass index, *dCBD* distal common bile duct, *AoV* ampulla of Vater; *LN* lymph node; *CR-POPF* clinically-relevant postoperative pancreatic fistula

^a^Median (Interquartile range)

^b^Clavien–Dindo grade ≥ IIIa

^c^Patients with lymph node metastasis

Clinical characteristics of the cohort that surpassed the learning curve are presented in Table 2. Of the 717 patients, 558 (77.8%) underwent open PD, and 159 (22.2%) received minimally invasive PD. There were no significant differences in baseline demographics, BMI, or, body composition measurements between the open PD and minimally invasive PD groups. The median operation time in the minimally invasive PD group was slightly longer than in the open PD group (295 versus 285 min, *P* = 0.050), but this difference was smaller compared to that observed in the entire cohort. The median intraoperative blood loss was significantly lower in the minimally invasive PD group than in the open PD group (340 versus 400 mL, *P* = 0.026). Additionally, unlike in the entire cohort, there were no significant differences in complication rates between the two groups (*P* = 0.859). The median length of hospital stay remained significantly shorter in the minimally invasive PD group than in the open PD group (8 versus 11 days, *P* < 0.001).

**Table 2:** Clinical characteristics according to the operation method in the cohort that surpassed the learning curve

Parameter	Total	Open	Robot	P
N (%)	717 (100.0)	558 (77.8)	159 (22.2)	
Age (years)^a^	68 (61–75)	68 (61–75)	66 (61–74)	0.166
Sex				0.973
Male	445 (62.1)	347 (62.2)	98 (61.6)	
Female	272 (37.9)	211 (37.8)	61 (38.4)	
ASA				0.170
I/II	629 (87.7)	484 (86.7)	145 (91.2)	
III/IV	88 (12.3)	74 (13.3)	14 (8.8)	
BMI (kg/m^2^)				0.391
< 25	515 (71.8)	396 (71.0)	119 (74.8)	
≥ 25	202 (28.2)	162 (29.0)	40 (25.2)	
Sarcopenia	376 (52.4)	291 (52.2)	85 (53.5)	0.840
Sarcopenic obesity	287 (40.0)	228 (40.9)	59 (37.1)	0.447
Diagnosis				< 0.001
Pancreas	259 (36.1)	215 (38.5)	44 (27.7)	
dCBD	262 (36.6)	213 (38.2)	49 (30.8)	
AoV	168 (23.4)	108 (19.4)	60 (37.7)	
Duodenum	28 (3.9)	22 (3.9)	6 (3.8)	
Operation time (min)^a^	290 (240–335)	285 (235–340)	295 (268–325)	0.050
Blood loss (mL)^a^	400 (240–560)	400 (250–600)	340 (185–515)	0.026
Harvested LN	18 (13–25)	19 (13–26)	18 (13–25)	0.312
LN metastasis	364 (50.8)	305 (54.7)	59 (37.1)	< 0.001
Resection margin				0.084
R0	552 (77.0)	421 (75.4)	131 (82.4)	
R1	165 (23.0)	137 (24.6)	28 (17.6)	
Complication^b^	143 (19.9)	110 (19.7)	33 (20.8)	0.859
CR-POPF	80 (11.2)	64 (11.5)	16 (10.1)	0.723
Hospital stays (days)	11 (8–16)	11 (9–18)	8 (7–10)	< 0.001
Adjuvant chemotherapy^c^	304 (83.5)	251 (82.3)	53 (89.3)	0.216

Values in parentheses are percentages unless indicated otherwise

*ASA* American Society of Anesthesiologists; *BMI* body mass index; *dCBD* distal common bile duct; *AoV* ampulla of Vater; *LN* lymph node; *CR-POPF* clinically relevant postoperative pancreatic fistula

^a^Median (Interquartile range)

^b^Clavien–Dindo grade ≥ IIIa

^c^Patients with lymph node metastasis

These findings align with previous studies suggesting that minimally invasive PD, when performed by experienced surgeons, can be as safe as open PD.

### Predictive factors for short-term surgical outcomes

To identify predictive factors for postoperative complications, univariate and multivariate logistic regression analyses were performed (Table 3). In the entire cohort, male sex, poor physical status, sarcopenic obesity (OR [95% CI]: 1.75 [1.24–2.46]; *P* = 0.001), distal common bile duct cancer (compared to pancreatic cancer), and minimally invasive PD (OR [95% CI]: 1.49 [1.04–2.11]; *P* = 0.028) were significantly associated with higher complication rates in the multivariate analysis. In the cohort that surpassed the learning curve, male sex, poor physical status, sarcopenic obesity (OR [95% CI]: 1.84 [1.23–2.77]; *P* = 0.003), and distal common bile duct cancer (compared to pancreatic cancer) remained independently associated with increased complication rates. However, surgical approach was not an independent predictor of complications in this cohort, unlike in the overall analysis. Additionally, high BMI and sarcopenia were not associated with postoperative complications in either cohort.

**Table 3:** Predictive factors for postoperative complications

Variables	Univariate analysis		Multivariate analysis	
	OR (95% CI)	*P*	OR (95% CI)	*P*
Entire cohort				
Age (years)				
≤ 65 vs. > 65	0.94 (0.69, 1.29)	0.703	NA	NA
Sex				
Female vs. male	1.82 (1.31, 2.57)	< 0.001	1.67 (1.19, 2.38)	0.004
ASA classification				
I/II vs. III/IV	1.97 (1.28, 2.97)	0.002	2.05 (1.32, 3.16)	0.001
BMI (kg/m^2^)				
< 25 vs. ≥ 25	1.38 (0.98, 1.92)	0.059	1.14 (0.79, 1.65)	0.475
Sarcopenia				
N vs. Y	1.09 (0.80, 1.49)	0.577	NA	NA
Sarcopenic obesity				
N vs. Y	1.92 (1.41, 2.63)	< 0.001	1.75 (1.24, 2.46)	0.001
Diagnosis				
Pancreas vs. dCBD	2.41 (1.66, 3.55)	< 0.001	2.18 (1.48, 3.24)	< 0.001
Pancreas vs. AoV	1.47 (0.95, 2.27)	0.083	1.36 (0.87, 2.13)	0.181
Pancreas vs. duodenum	1.63 (0.66, 3.62)	0.255	1.36 (0.54, 3.10)	0.486
Method				
Open vs. robot	1.35 (0.97, 1.89)	0.076	1.49 (1.04, 2.11)	0.028
Harvested LN				
< 17 vs. ≥ 17	1.21 (0.86, 1.71)	0.280	NA	NA
LN metastasis				
N vs. Y	0.92 (0.68, 1.26)	0.612	NA	NA
Cohort after learning curve				
Age (years)				
≤ 65 vs. > 65	0.96 (0.66, 1.39)	0.825	NA	NA
Sex				
Female vs. male	1.99 (1.33, 3.02)	0.001	1.82 (1.20, 2.79)	0.005
ASA classification				
I/II vs. III/IV	1.96 (1.18, 3.19)	0.008	1.89 (1.12, 3.14)	0.015
BMI (kg/m^2^)				
< 25 vs. ≥ 25	1.27 (0.85, 1.88)	0.236	1.00 (0.64, 1.55)	0.995
Sarcopenia				
N vs. Y	1.07 (0.74, 1.55)	0.707	NA	NA
Sarcopenic obesity				
N vs. Y	1.95 (1.35, 2.83)	< 0.001	1.84 (1.23, 2.77)	0.003
Diagnosis				
Pancreas vs. dCBD	2.65 (1.71, 4.20)	< 0.001	2.40 (1.53, 3.83)	< 0.001
Pancreas vs. AoV	1.38 (0.80, 2.36)	0.241	1.38 (0.79, 2.39)	0.258
Pancreas vs. duodenum	1.44 (0.46, 3.77)	0.490	1.20 (0.37, 3.26)	0.739
Method				
Open vs. robot	1.07 (0.68, 1.64)	0.772	NA	NA
Harvested LN				
< 17 vs. ≥ 17	1.14 (0.76, 1.74)	0.547	NA	NA
LN metastasis				
N vs. Y	0.98 (0.68, 1.41)	0.911	NA	NA

*NA* not available, *ASA* American Society of Anesthesiologists, *BMI* body mass index, *dCBD* distal common bile duct, *AoV* ampulla of Vater, *LN* lymph node

**Fig. 1:**
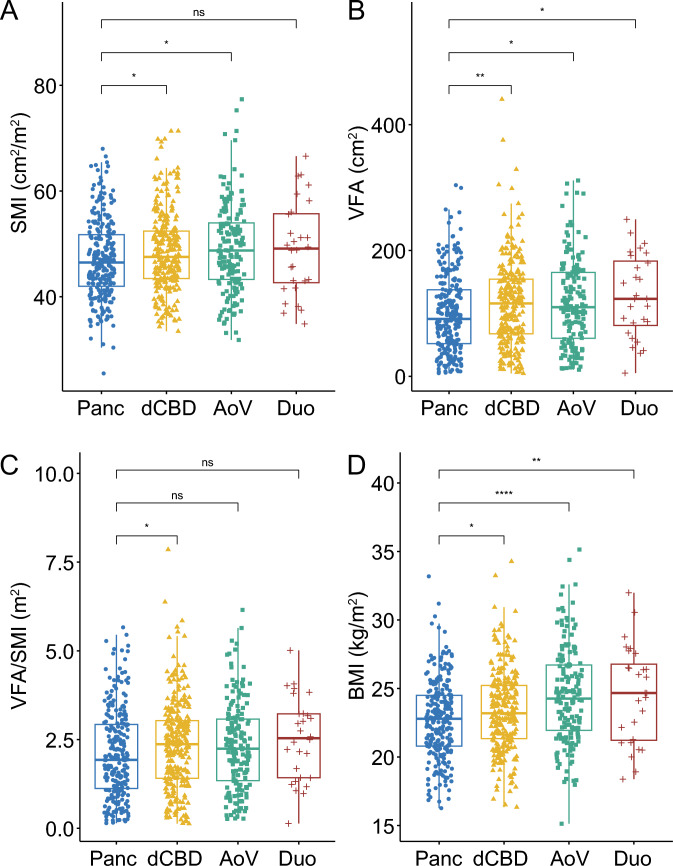
Skeletal muscle index (**A**), visceral fat area (**B**), visceral fat area to skeletal muscle index ratio (**C**), and body mass index (**D**) according to the cancer origin. *SKI* skeletal muscle index, *Panc* pancreas, *dCBD* distal common bile duct, *AoV* ampulla of Vater, *Duo* duodenum, *VFA* visceral fat area, *BMI* body mass index

In the cohort that surpassed the learning curve, body composition metrics and BMI were compared across cancer types (Fig. 1). Patients with distal common bile duct and ampulla of Vater cancers exhibited significantly higher median SMI, VFA, and BMI values than those with pancreatic cancer. However, with respect to the VFA/SMI ratio, only patients with distal common bile duct cancer (2.4 m^2^) had a significantly higher median value than those with pancreatic cancer (1.9 m^2^). These findings are consistent with the logistic regression analysis, which demonstrated that, compared to pancreatic cancer, only distal common bile duct cancer was independently associated with increased complication rates.

### Subgroup analyses according to the sarcopenic obesity

Based on the logistic regression analyses, the cohort that surpassed the learning curve was stratified according to the presence or absence of sarcopenic obesity. Clinical characteristics of patients without sarcopenic obesity are presented in Table S1. Complication rates (15.5% versus 16.0%, *P* > 0.99) and clinically relevant postoperative pancreatic fistula rates (6.7% versus 7.0%, *P* > 0.99) were comparable between the open and minimally invasive PD groups. The median length of hospital stay was significantly shorter in the minimally invasive PD group than in the open PD group (8 versus 11 days, *P* < 0.001). Additionally, in terms of short-term oncologic feasibility, the two groups had a comparable number of harvested lymph nodes and similar R0 resection rates.

Clinical characteristics of patients with sarcopenic obesity are presented in Table 4. There were no significant differences in sex, physical status, or cancer type between the open and minimally invasive PD groups. Complication rates (25.9% versus 28.8%, *P* = 0.772) and clinically relevant postoperative pancreatic fistula rates (18.4% versus 15.3%, *P* = 0.707) were also not significantly different between the groups; however, all these rates were higher than those observed in patients without sarcopenic obesity. The median length of hospital stay was significantly shorter in the minimally invasive PD group than in the open PD group (8 versus 13 days, *P* < 0.001). As with the non-sarcopenic obesity group, short-term oncologic outcomes—including the number of harvested lymph nodes and R0 resection rates—were comparable between the two groups.

**Table 4:** Clinical characteristics according to the operation method in patients with sarcopenic obesity among the cohort that surpassed the learning curve

Parameter	Open	Robot	*P*
*N*	228	59	
Age (years)^a^	70 (63–76)	70 (62–77)	0.971
Sex (male)	149 (65.4)	37 (62.7)	0.822
ASA			0.630
I/II	197 (86.4)	53 (89.8)	
III/IV	31 (13.6)	6 (10.2)	
BMI (kg/m^2^) ≥ 25	114 (50.0)	25 (42.4)	0.369
Diagnosis			0.236
Pancreas	73 (32.0)	16 (27.1	
dCBD	96 (42.1)	20 (33.9)	
AoV	48 (21.1)	20 (33.9)	
Duodenum	11 (4.8)	3 (5.1)	
Operation time (min)^a^	285 (240–360)	300 (270–328)	0.353
Blood loss (mL)^a^	435 (300–700)	450 (265–560)	0.483
Harvested LN	17 (11–24)	15 (11–23)	0.419
LN metastasis	115 (50.4)	22 (37.3)	0.098
Resection margin			0.351
R0	174 (76.3)	49 (83.1)	
R1	54 (23.7)	10 (16.9)	
Complication^b^	59 (25.9)	17 (28.8)	0.772
CR-POPF	42 (18.4)	9 (15.3)	0.707
Hospital stays (days)	13 (9–21)	8 (7–12)	< 0.001
Adjuvant chemotherapy^c^	96 (83.5)	20 (90.9)	0.527

Values in parentheses are percentages unless indicated otherwise

*ASA* American Society of Anesthesiologists, *BMI* body mass index, *dCBD* distal common bile duct, *AoV* ampulla of Vater, *LN* lymph node, *CR-POPF* clinically-relevant postoperative pancreatic fistula

^a^Median (Interquartile range)

^b^Clavien–Dindo grade ≥ IIIa

^c^Patients with lymph node metastasis

### Predictive factors for long-term oncologic outcomes

To identify predictive factors for recurrence, univariate and multivariate Cox proportional hazards regression analyses were performed in patients who underwent long-term follow-up (> 5 years) from the cohort that surpassed the learning curve (Table S2). In multivariate analysis, poor physical status, pancreatic cancer (compared to distal common bile duct cancer or ampulla of Vater cancer), and lymph node metastasis were identified as independent risk factors for recurrence. Minimally invasive PD, however, was not significantly associated with increased recurrence rates. With respect to BMI and body composition factors, neither high BMI nor sarcopenic obesity was associated with recurrence. Sarcopenia, however, was marginally associated with recurrence (HR [95% CI]: 1.32 [0.97–1.78]; *P* = 0.076).

## Discussion

Although the clinical utility of body composition assessment in patients with cancer has received considerable attention and has been extensively studied, few studies have focused on periampullary cancer. As research on body composition has primarily related to surgical complexity, the relatively late adoption of minimally invasive PD—compared to other abdominal procedures—may partly explain the lack of evidence in this field. Our institution had the advantage of being one of the most experienced centers globally in performing minimally invasive PD at the time of this study [15]. This study demonstrated that sarcopenic obesity negatively impacts short-term surgical outcomes, and that sarcopenia may be associated with worse long-term oncologic outcomes. In contrast, high BMI was not a reliable predictor of either short- or long-term postoperative outcomes. Furthermore, this study confirmed that minimally invasive PD can be performed safely as open PD—even in patients with sarcopenic obesity—when conducted by experienced surgeons.

Due to the growing prevalence of obesity as a global health issue, the clinical impact of high BMI in patients with periampullary cancer has been investigated in several previous studies. However, their findings have been inconsistent. Regarding short-term outcomes, Li et al. (2024) reported no significant differences in clinically relevant complications after laparoscopic PD between overweight (BMI > 24 kg/m^2^) and normal-weight groups (*P* = 0.410), whereas Chao et al. (2021) identified obesity (BMI > 27.5 kg/m^2^) as the only independent predictor of clinically relevant complications after robotic PD [27, 28]. Additionally, both Napoli et al. (2023) and Peng et al. (2023) developed difficulty scoring systems for minimally invasive PD, but only Napoli et al. (2023) included high BMI (≥ 25 kg/m^2^ for males, ≥ 30 kg/m^2^ for females) as a scoring variable [29, 30]. There are two plausible explanations for these inconsistent findings. First, considerable variation in patient BMI and surgeon experience with minimally invasive PD exists across regions and medical centers. Moreover, the aforementioned studies applied different BMI cutoff values. Second, BMI alone may not serve as a reliable predictor of short-term surgical outcomes. The primary intraoperative challenge is difficulty in identifying anatomic landmarks and dissection planes due to visceral fat. Additionally, secretion of proinflammatory cytokines is increased in response to increased body fat tissue, resulting in a chronic inflammatory state that impedes wound healing and physical recovery following surgery [31]. Accordingly, in our study, sarcopenic obesity—which incorporates the effect of visceral fat—was identified as an independent predictor of postoperative complications. These patients may benefit from enhancing perioperative care after identifying sarcopenic obesity and precisely understanding the anatomic structure using 3-dimensional reconstruction of preoperative images. Regarding long-term outcomes, Cui et al. (2022) demonstrated that a high BMI (≥ 25 kg/m^2^) was associated with improved overall survival following PD for periampullary cancer, whereas Seika et al. (2019) reported no significant differences in overall survival among normal-weight (18.5 ≤ BMI ≤ 25.0 kg/m^2^), overweight (25.0 < BMI ≤ 30.0 kg/m^2^), and obese (BMI > 30.0 kg/m^2^) groups [32, 33]. A recent meta-analysis of patients with pancreatic cancer found that sarcopenia was a negative prognostic factor in both curative and palliative treatment settings [34]. Additionally, Min et al. (2025) reported that sarcopenic deterioration during chemotherapy was associated with poor survival outcomes in patients with pancreatic cancer [35]. In our study, neither BMI nor sarcopenic obesity was associated with recurrence, whereas sarcopenia was. These findings suggest that evaluating body composition—rather than BMI alone—may be more useful for predicting both short- and long-term outcomes after surgery in patients with periampullary cancer. However, as most studies on the association between body composition profile and long-term outcomes are small-sized retrospective studies and some studies reported no association between the two, so this finding should be interpreted with caution [36].

Another important finding of our study is that minimally invasive PD may be as safe as open PD in patients with sarcopenic obesity when performed by experienced surgeons. Many novice surgeons encounter difficulties in identifying anatomic landmarks and dissection planes, particularly in patients with substantial visceral fat, which complicates the procedure. These challenges are amplified in minimally invasive PD due to limitations in tactile feedback, such as palpating major arteries or directly controlling bleeding—capabilities more readily available in open PD. However, such technical challenges can be overcome through sufficient procedural experience. Recent studies on simulation-based training and mentorship programs aimed at reducing the duration of the learning curve are gaining attention [37, 38]. Based on the findings of our study, future training curricula should consider incorporating patient-specific factors—such as sarcopenic obesity—that may increase surgical complexity.

A key strength of this study is that it evaluated the clinical significance of sarcopenia and sarcopenic obesity in patients with periampullary cancer. Moreover, the procedures were conducted at one of the most experienced institutions globally for minimally invasive PD. Nevertheless, several limitations should be acknowledged. First, our institution is a high-volume, academic, pancreatic surgery specialty center; therefore, these findings may not be generalizable to lower-volume or non-academic centers. Second, as this is a retrospective cohort study, issues like selection bias, including various cancer types, are inherent to the study. Third, because the Republic of Korea has a relatively low prevalence of morbid obesity (BMI > 30 kg/m^2^), we were unable to perform subgroup analyses on higher BMI thresholds. Fourth, we did not assess factors like nutritional status or frailty index that are thought to be significant in addition to body composition. Because these limitations make external application of these results difficult, future international, multicenter, prospective clinical trials are warranted to validate these findings.

## Conclusion

This study demonstrated that computed tomography-derived body composition assessment is clinically valuable in patients with resected periampullary cancer. Sarcopenic obesity was associated with adverse short-term surgical outcomes. Additionally, we found that minimally invasive PD can be carefully performed by surgeons who have surpassed the learning curve even in patients with sarcopenic obesity. These findings may not only offer clinical benefit to patients with periampullary cancer but also assist in patient selection for surgeons in the early stages of adopting minimally invasive PD.

## Supplementary Information

Below is the link to the electronic supplementary material.

Supplementary file1 (DOCX 337 KB)
