# Supplementary material for: Association of computed tomography-derived body composition with surgical and oncologic outcomes in periampullary adenocarcinoma
Source: Surg Endosc. 2026 Feb 6;40(4):3330–40. doi: 10.1007/s00464-026-12601-2 (PMC13053584; doi:10.1007/s00464-026-12601-2)
Supplement: Supplementary file 1 — Supplementary file1 (DOCX 337 KB) [file 464_2026_12601_MOESM1_ESM.docx]

**Fig. S1** Flow diagram of this study


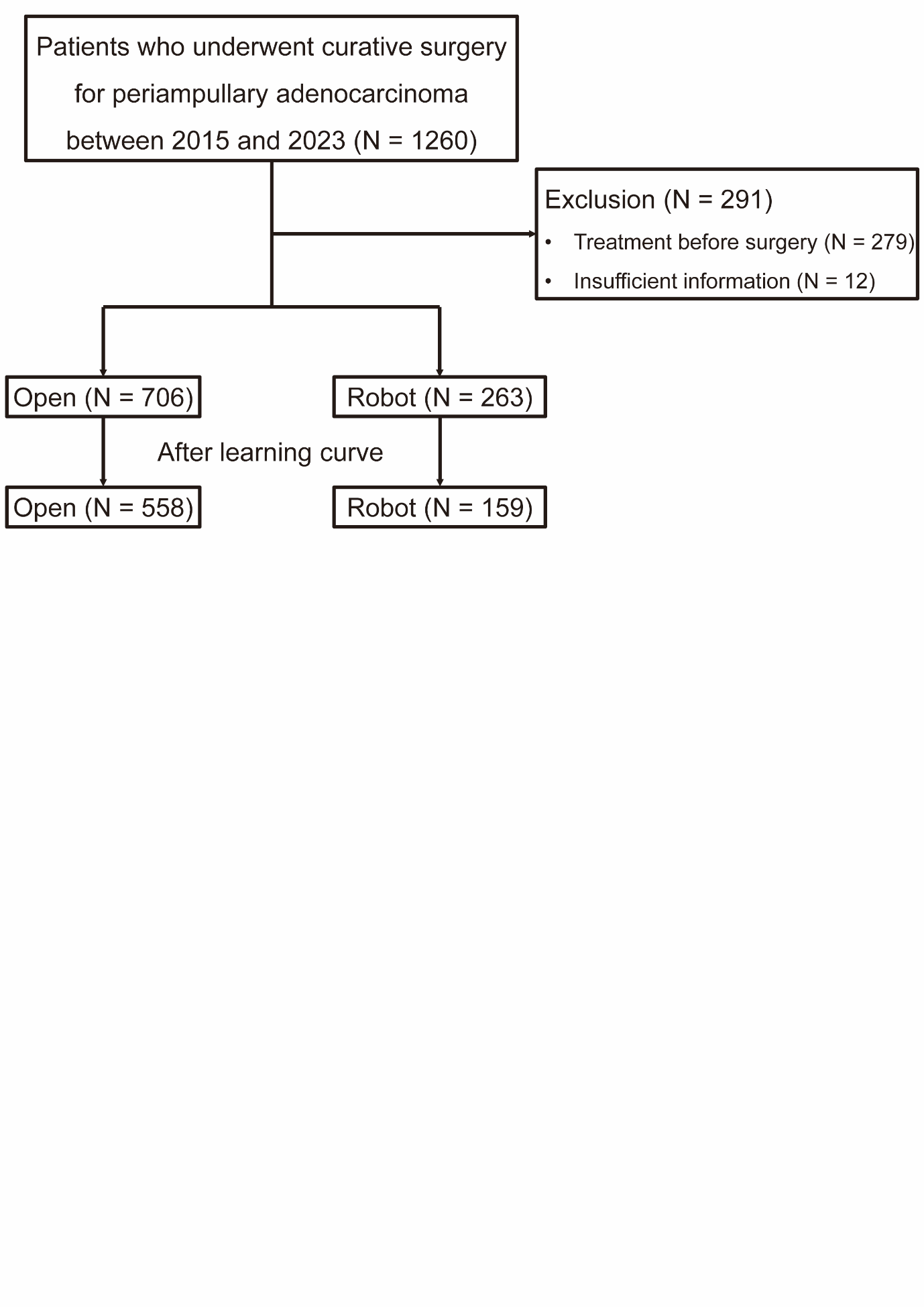


**Table S1** Clinical characteristics according to the operation method in patients without sarcopenic obesity among the cohort that surpassed the learning curve

| **Parameter** | **Open** | **Robot** | **P** |
| --- | --- | --- | --- |
|  |  |  |  |
| N (%) | 330 | 100 |  |
| Age (years)^*^ | 67 (60–74) | 65 (60–71) | 0.176 |
| Sex (Male) | 198 (60.0) | 61 (61.0) | 0.950 |
| ASA |  |  | 0.236 |
| I / II | 287 (87.0) | 92 (92.0) |  |
| III / IV | 43 (13.0) | 8 (8.0) |  |
| BMI (kg/m^2^) ≥ 25 | 48 (14.5) | 15 (15.0) | >0.99 |
| Diagnosis |  |  | <0.001 |
| Pancreas | 142 (43.0) | 28 (28.0) |  |
| dCBD | 117 (35.5) | 29 (29.0) |  |
| AoV | 60 (18.2) | 40 (40.0) |  |
| Duodenum | 11 (3.3) | 3 (3.0) |  |
| Operation time (min)^*^ | 285 | 293 | 0.077 |
|  | (230–335) | (264–325) |  |
| Blood loss (mL)^*^ | 350 | 255 | 0.023 |
|  | (200–500) | (150–493) |  |
| Harvested LN | 20 (14–27) | 19 (14–25) | 0.449 |
| LN metastasis | 190 (57.6) | 37 (37.0) | <0.001 |
| Resection margin |  |  | 0.179 |
| R0 | 247 (74.8) | 82 (82.0) |  |
| R1 | 83 (25.2) | 18 (18.0) |  |
| Complication^§^ | 51 (15.5) | 16 (16.0) | >0.99 |
| CR-POPF | 22 (6.7) | 7 (7.0) | >0.99 |
| Hospital stays (days) | 11 (9–15) | 8 (7–10) | <0.001 |
| Adjuvant chemotherapy^†^ | 155 (81.6) | 33 (89.2) | 0.376 |

Values in parentheses are percentages unless indicated otherwise

^a^Median (Interquartile range)

^b^Clavien-Dindo grade ≥ IIIa

^c^Patients with lymph node metastasis

*ASA* American Society of Anesthesiologists; *BMI* body mass index; *dCBD* distal common bile duct; *AoV* ampulla of vater; *LN* lymph node; *CR-POPF* clinically-relevant postoperative pancreatic fistula

**Table S2** Risk factors for recurrence in patients who underwent long-term follow up among the cohort that surpassed the learning curve

| **Variables** | **Univariate analysis** | | **Multivariate analysis** | |
| --- | --- | --- | --- | --- |
|  | **OR (95% CI)** | **P** | **OR (95% CI)** | **P** |
| Age (years) |  |  |  |  |
| ≤ 65 vs. > 65 | 1.06 (0.80, 1.41) | 0.669 | NA | NA |
| Sex |  |  |  |  |
| Female vs. Male | 0.93 (0.70, 1.24) | 0.617 | NA | NA |
| ASA classification |  |  |  |  |
| I / II vs. III / IV | 1.32 (0.89, 1.97) | 0.173 | 1.61 (1.06, 2.44) | 0.026 |
| BMI (kg/m^2^) |  |  |  |  |
| < 25 vs. ≥ 25 | 0.78 (0.56, 1.07) | 0.124 | 0.93 (0.64, 1.33) | 0.673 |
| Sarcopenia |  |  |  |  |
| N vs. Y | 1.25 (0.94, 1.65) | 0.120 | 1.32 (0.97, 1.78) | 0.076 |
| Sarcopenic obesity |  |  |  |  |
| N vs. Y | 1.02 (0.77, 1.36) | 0.871 | NA | NA |
| Diagnosis |  |  |  |  |
| Pancreas vs. dCBD | 0.41 (0.30, 0.58) | <0.001 | 0.53 (0.37, 0.75) | <0.001 |
| Pancreas vs. AoV | 0.28 (0.18, 0.45) | <0.001 | 0.52 (0.31, 0.88) | 0.014 |
| Pancreas vs. Duodenum | 0.92 (0.50, 1.71) | 0.790 | 1.06 (0.55, 2.07) | 0.854 |
| Method |  |  |  |  |
| Open vs. Robot | 0.69 (0.34, 1.41) | 0.312 | NA | NA |
| Harvested LN |  |  |  |  |
| < 17 vs. ≥ 17 | 1.39 (0.99, 1.95) | 0.057 | 1.22 (0.86, 1.72) | 0.268 |
| LN metastasis |  |  |  |  |
| N vs. Y | 2.74 (2.02, 3.71) | <0.001 | 1.82 (1.33, 2.50) | <0.001 |
| Resection margin |  |  |  |  |
| R0 vs. R1 | 1.83 (1.36, 2.46) | <0.001 | 1.15 (0.82, 1.60) | 0.416 |
| Adjuvant chemotherapy |  |  |  |  |
| N vs. Y | 5.40 (3.28, 8.88) | <0.001 | 3.57 (2.13, 5.98) | <0.001 |

*ASA* American Society of Anesthesiologists; *BMI* body mass index; *dCBD* distal common bile duct; *AoV* ampulla of vater; *LN* lymph node
